# Novel Epidemic Clones of *Listeria monocytogenes*, United States, 2011

**DOI:** 10.3201/eid1901.121167

**Published:** 2013-01

**Authors:** Sara Lomonaco, Bindhu Verghese, Peter Gerner-Smidt, Cheryl Tarr, Lori Gladney, Lavin Joseph, Lee Katz, Maryann Turnsek, Michael Frace, Yi Chen, Eric Brown, Richard Meinersmann, Mark Berrang, Stephen Knabel

**Affiliations:** Author affiliations: Università degli Studi di Torino, Turin, Italy (S. Lomonaco);; Accugenix, Newark, Delaware, USA (B. Verghese);; The Pennsylvania State University, University Park, Pennsylvania, USA (B. Verghese, S. Knabel);; Centers for Disease Control and Prevention, Atlanta, Georgia, USA (P. Gerner-Smidt, C. Tarr, L. Gladney, L. Joseph, L. Katz, M. Turnsek, M. Frace);; Food and Drug Administration, College Park, Maryland, USA (Y. Chen, E. Brown);; US Department of Agriculture, Athens, Georgia, USA (R. Meinersmann, M. Berrang)

**Keywords:** *Listeria monocytogenes*, cantaloupe, United States, novel outbreak strain, novel epidemic clones, mixed-serotype biofilms, bacteria, foodborne infections

## Abstract

We identified a novel serotype 1/2a outbreak strain and 2 novel epidemic clones of *Listeria monocytogenes* while investigating a foodborne outbreak of listeriosis associated with consumption of cantaloupe during 2011 in the United States. Comparative analyses of strains worldwide are essential to identification of novel outbreak strains and epidemic clones.

In September 2011, the Centers for Disease Control and Prevention (CDC) in Atlanta, GA, was notified of an increase of listeriosis cases linked to eating cantaloupe (*1*). The outbreak isolates were categorized into 4 pulsed-field gel electrophoresis (PFGE) profiles and serotypes 1/2a and 1/2b, the latter being seldom associated with large outbreaks (*1**,**2*). During August 2012, a fifth outbreak-associated subtype responsible for 1 case was detected, and CDC reported a final total of 147 cases from 28 US states, causing 33 deaths and 1 miscarriage (www.cdc.gov/listeria/outbreaks/cantaloupes-jensen-farms/index.html). The Food and Drug Administration (FDA) inspected the involved farm; outbreak strains matching 3 of the PFGE profiles from clinical samples were isolated from washed cantaloupes and various environmental surfaces within the facility (www.fda.gov/Food/FoodSafety/CORENetwork/ucm272372.htm#report).

Epidemic clones (ECs) of *Listeria monocytogenes* are defined as isolates of a presumably common ancestor that are genetically related and involved in different temporally and geographically unrelated outbreaks (*2*). Previously, multivirulence locus sequence typing (MVLST) accurately identified the 5 known ECs of *L. monocytogenes*, ECI–V (*3**,**4*). Also, *comK* prophage junction fragment (JF) sequences were demonstrated to be unique to EC strains of *L. monocytogenes* in individual facilities that processed ready-to-eat meat and poultry or in multiple plants manufacturing similar ready-to-eat products (*5*). The *comK* prophage may represent a rapid adaptation island that enables *L. monocytogenes* to rapidly adapt to and form biofilms in specific environmental niches (*5*).

Nine foodborne outbreak-associated isolates related to cantaloupe, representing the 4 outbreak strains initially identified, were selected for multilocus sequence typing (MLST) (*6*), MVLST (*3*), and *comK* prophage JF sequencing (*5*) to determine if they represented previously identified outbreak strains or known/novel ECs of *L. monocytogenes* (*2*–*4*). Isolates from cantaloupe samples were also compared with 29 US Department of Agriculture (USDA) isolates of *L. monocytogenes* retrieved from 2 US chicken processing plants (*7**,**8*).

## The Study

**Figure 1:**
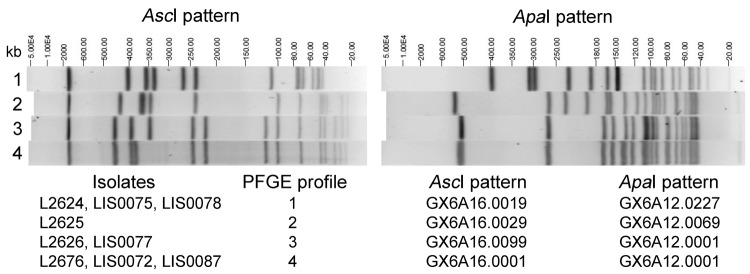
Four *Asc*I/*Apa*I pulsed-field gel electrophoresis (PFGE) profiles (identified at the time the research was performed) displayed by *Listeria monocytogenes* clinical isolates (L2624, L2625, L2626, and L2676) and isolates from food or environmental samples (LIS0072, LIS0075, LIS0077, LIS0078, and LIS0087) associated with the 2011 listeriosis outbreak traced to cantaloupe. PFGE profiles 3 and 4 differ by ≈40-kb shift in 1 band in the *Asc*I pattern, likely related to the loss or acquisition of the *comK* prophage, because the size of this prophage was ≈40 kb as calculated by using the whole genome sequencing data (not shown).

CDC confirmed identification of *L. monocytogenes* using the AccuProbe LISTERIA MONOCYTOGENES Culture Identification Test (Gen-Probe, San Diego, CA, USA) and by FDA according to the FDA Bacteriological Analytical Manual (www.fda.gov/Food/ScienceResearch/LaboratoryMethods/BacteriologicalAnalyticalManualBAM/default.htm). Isolates were serotyped by using commercial antisera (Denka Seiken, Tokyo, Japan) and analyzed by PFGE (*9*) (Table; Figure 1). The Technical Appendix shows the relative distribution of the 4 PFGE profiles among clinical, food, or environmental samples.

**Table:** Characteristics of *Listeria monocytogenes* isolates representing 1 novel outbreak strain and 2 newly defined epidemic clones, ECVI and ECVII, United States, 2011*

Isolate†	Agency	Outbreak year, location, source (type of source)	Serotype	MLST ST (CC)	MVLST VT (EC)	UP PT	DOWN PT
PFGE profile1‡							
L2624	CDC	2011, US, cantaloupe (C)	1/2b	5 (5)	63 (VI)	–	–
LIS0075	FDA	2011, US, cantaloupe (F)	1/2b	5 (5)	63 (VI)	–	–
LIS0078	FDA	2011, US, cantaloupe (E)	1/2b	5 (5)	63 (VI)	–	–
233	USDA	2002, US, chicken plant A (F)	1/2b	5 (5)	63 (VI)	–	–
466	USDA	2006, US, chicken plant B (F)	1/2b	5 (5)	63 (VI)	–	11
10–0810	NML	1996, Canada, imitation crabmeat (C)	1/2b	5 (5)	63 (VI)	11	11
10–0811	NML	1996, Canada, imitation crabmeat (F)	1/2b	5 (5)	63 (VI)	11	11
PFGE profile 2							
L2625	CDC	2011, US, cantaloupe (C)	1/2a	29 (29)	74	–	–
PFGE profile 3							
L2626	CDC	2011, US cantaloupe (C)	1/2a	561 (7)§	56 (VII)	–	–
LIS0077	FDA	2011 US, cantaloupe (E)	1/2a	561 (7)	56 (VII)	–	–
PFGE profile 4							
L2676	CDC	2011, US, cantaloupe (C)	1/2a	7 (7)	56 (VII)	18	13
LIS0072	FDA	2011, US, cantaloupe (F)	1/2a	7 (7)	56 (VII)	18	13
LIS0087	FDA	2011, US, cantaloupe (E)	1/2a	7 (7)	56 (VII)	18	13
261	USDA	2002, US, chicken plant A (E)	1/2a	7 (7)	56 (VII)	18	13
498	USDA	2006, US, chicken plant B (E)	1/2a	7 (7)	56 (VII)	–	10
10–813	NML	2000 Canada, whipping cream (C)	1/2a	7 (7)	56 (VII)	–	13
10–812	NML	2000 Canada, whipping cream (F)	1/2a	7 (7)	56 (VII)	–	13

*For comparison, additional molecular subtype data from unrelated foodborne outbreaks in Canada were obtained (*4*). PFGE, pulsed-field gel electrophoresis; CDC, Centers for Disease Control and Prevention; FDA, Food and Drug Administration; USDA, US Department of Agriculture; NML, National Microbiology Laboratory of Canada, Division of the Public Health Agency of Canada; MLST ST (CC), multilocus sequence typing, sequence type (clonal complex); MVLST VT (EC), multivirulence locus sequence typing, virulence type (epidemic clone); UP PT, upstream *comK* prophage type; DOWN PT, downstream *comK* prophage type; –, no PCR amplification of junction fragment. †The numbering of outbreak strain used differs from the FDA’s final update on the outbreak (www.fda.gov/Food/FoodSafety/CORENetwork/ucm272372.htm#report). ‡PFGE profiles based on CDC and FDA analysis of isolates from cantaloupe-associated outbreak. §ST561 differs from ST7 by 1 nonsynonymous single nucleotide polymorphism (G/A) in *lhkA*.

Isolates were grown overnight in tryptic soy broth with yeast extract at 37°C, and DNA was extracted by using the Ultra Clean Microbial DNA Isolation Kit (Mo Bio Laboratories, Solana Beach, CA, USA) for isolates from CDC and USDA and the Wizard Genomic DNA Purification Kit (Promega, Madison, WI, USA) for isolates from FDA. Sequence types (STs) identified by using MLST were assigned as described (*6*) on the basis of whole genome sequence data (C. Tarr, Y. Chen, unpub. data) and compared with those publicly available (www.pasteur.fr/mlst). MVLST data were obtained as described (*3*) or extracted from whole genome sequences (Y. Chen, unpub. data). Sequences were compared with those on the MVLST database available in the laboratory of S.K. (*3**,**4*) and analyzed by using MEGA5.0 (*10*). New virulence types (VTs) were assigned to USDA isolates: VT60 (isolates 239, 441, 442, 458, 541, 565, 577); VT68 (350, 470); VT69 (247); VT70 (502); VT71 (450); VT72 (342), and VT73 (267). *comK* prophage JFs were sequenced as described (*5*). Prophage types (PTs) were assigned by comparing JF sequences with those available from previous reports (*4**,**5*). *comK* prophage JF sequences were submitted to GenBank for isolate L2676 (accession nos. JQ407079 and JQ407080) and 3 USDA isolates (accession nos. JQ750615–JQ750618).

**Figure 2:**
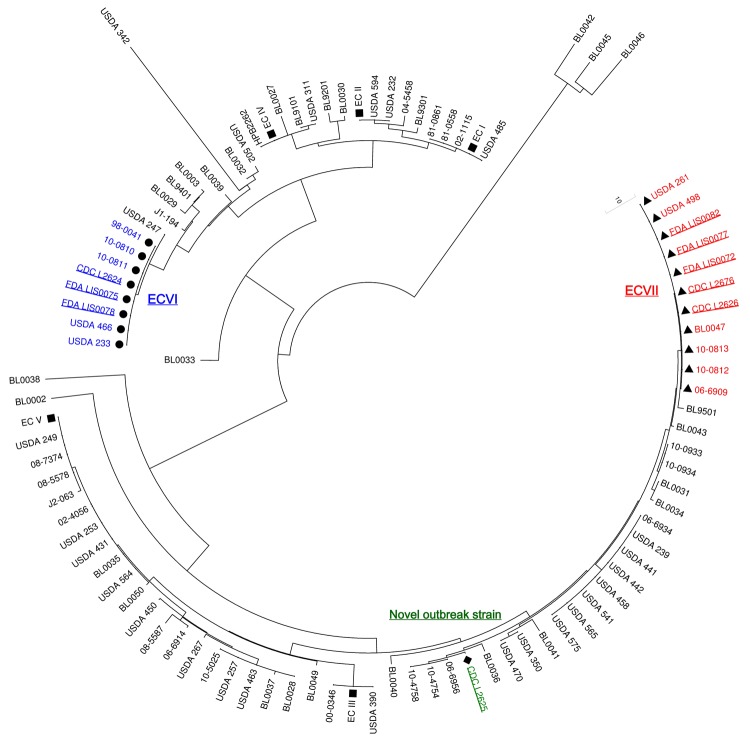
Unrooted neighbor-joining tree computed in MEGA 5.0 (*10*) for multivirulence locus sequence typing data based on sequencing of 6 virulence genes, *prfA*, *inlB*, *inlC*, *dal*, *clpP*, and *lisR* (*3*) obtained for the 93 *Listeria monocytogenes* isolates compared in this study. Nine cantaloupe-associated outbreak isolates were from the Centers for Disease Control and Prevention (CDC) (GenBank accession nos. JQ407055–JQ407078) and the Food and Drug Administration (FDA) (JX141237–JX141275), 23 isolates from Knabel et al., 2012 (*4*), 29 isolates from Chen et al., 2007 (*3*), 29 from Berrang et al., 2005 and 2010 (*7**,**8*) (JQ946653–JQ946836), and 3 other isolates for which sequence data were available in GenBank. Squares indicate reference strains representing the 5 currently known *L. monocytogenes* epidemic clones. Underlined text indicates CDC and FDA isolates associated with the isolates from the 2011 cantaloupe-associated outbreak. Circles indicate strains classified as epidemic clone (EC)VI (indicated in blue). Triangles indicate strains classified as ECVII (indicated in red). Diamond indicates the novel outbreak strain (indicated in green).

Isolates L2624, LIS0075, and LIS0078 (PFGE profile 1) belonged to the globally disseminated ST5 (*6*) and had the same VT (VT63) as 5 other 1/2b isolates in the database: isolates 10-0810 and 10-0811, from an imitation crabmeat–borne outbreak in Canada during 1996 (*4**,**11*); and isolates 98-0041, 233, and 466 (Table; Figure 2). Because VT63 isolates were associated with multiple outbreaks, they should be considered part of a novel EC (ECVI). ECVI isolates from cantaloupe and USDA isolate 233 showed no amplification of *comK* prophage JFs (Table). PT11/11 was identified during the 1996 imitation crabmeat–associated outbreak in Canada (*4*) and in USDA isolate 466 (Table). Further research is needed to determine why *comK* PTs were identical during different years and in different geographic locations and food processing plants.

Isolate L2625 (VT74, PFGE profile 2) from cantaloupe differed by 1 single nucleotide polymorphism in *inlC* from 3 other serotype 1/2a VT61 isolates (10-4758, 10-4754, and 06-6956) associated with the 2002 cheese-associated listeriosis outbreak in Canada (*4**,**12*) (Table; Figure 2). L2625 was assigned to ST29, an infrequent sequence type in the Institut Pasteur MLST database that differs from the ST (ST405) assigned to the isolates from cheese in the 2002 outbreak in Canada. No amplification of *comK* prophage JFs was observed, consistent with the PTs in the 2002 cheese-associated outbreak in Canada (*4*). Given this evidence, isolate L2625 does not represent a novel EC but should be considered a novel outbreak strain.

Isolates L2626 and LIS0077 (PFGE profile 3, ST7) and L2676, LIS0072, and LIS0087 (PFGE profile 4, ST561) from cantaloupe samples shared the same VT (VT56) as isolates 10-0813 and 10-0812 associated with a listeriosis outbreak related to whipping cream during 2000 in Canada (*4**,**12*) and isolates 06-6909, BL0047, 261, and 498 (Table; Figure 2). These *Listeria* isolates from cantaloupe displayed 2 highly similar PFGE profiles and STs, and the same serotype, *Apa*I PFGE pattern, and VT (Table; Figure 1). Isolates L2626 and LIS0077 showed no amplification of *comK* prophage JFs, which was also consistent with the upstream PT in the outbreak associated with whipping cream in Canada (Table). The JF sequences in isolates L2676, LIS0072, and LIS0087 were identical to those in USDA isolate 261 (Table). These isolates matched those from the whipping cream–associated outbreak in Canada in terms of VT56 and downstream PT (PT13) (Table). However, the upstream JF could not be amplified in the strain identified in whipping cream (*4*), possibly because of extensive recombination within the *comK* prophage (*13*), especially in the upstream JF (*5*). These STs and VTs were also found in clinical isolates over extended periods (*6*). Therefore, by definition (*2**,**3*), these isolates also represent a novel EC (ECVII).

## Conclusions

Different clones, particularly ECVI and ECVII, might have cocolonized niches or harborage sites within the cantaloupe processing facility, possibly explaining the multiple strains associated with this outbreak. Serotype 4b *L. monocytogenes* strains, of the same genetic lineage as serotype 1/2b strains, reportedly survived and grew substantially better in mixed-serotype biofilms containing a specific strain of serotype 1/2a (*14*). Although a biofilm was not detected in the cantaloupe facility, because the facility had already been extensively cleaned and sanitized before FDA sampling, further research is needed to determine the potential for these strains to cocolonize with biofilms.

Six of the 7 currently identified ECs were found at some point in 1 or both of the US chicken processing plants included in the study (Figure 2). Listeriosis cases and outbreaks have been associated with consumption of undercooked raw chicken and ready-to-eat poultry products (*2**,**4*). Additional research is needed to determine whether poultry or poultry processing plants could be responsible for the global dissemination of ECs of *L. monocytogenes*.

The molecular epidemiology of *L. monocytogenes* strains involved in the 2011 multistate cantaloupe-associated outbreak was greatly enhanced by the use of subtyping markers with different levels of epidemiologic resolution. Particularly, MVLST enabled the detection of 1 novel 1/2a outbreak strain and 2 novel ECs of *L. monocytogenes*. In contrast to focusing on isolates from a single outbreak (*15*), our findings demonstrate that to detect new ECs it is important to analyze isolates from many sources around the world.

Technical Appendix Number of isolates of *Listeria monocytogenes* encountered in clinical and food or environment samples collected by the Centers for Disease Control and Prevention during a 2011 *L. monocytogenes* outbreak related to cantaloupe.
