# Supplementary material for: Novel Epidemic Clones of Listeria monocytogenes, United States, 2011
Source: Emerg Infect Dis. 2013 Jan;19(1):147–50. doi: 10.3201/eid1901.121167 (PMC3558006; doi:10.3201/eid1901.121167)
Supplement: Technical Appendix — Number of isolates of Listeria monocytogenes encountered in clinical and food or environment samples collected by the Centers for Disease Control and Prevention during a 2011 L. monocytogenes outbreak related to cantaloupe. [file 12-1167-Techapp-s1.pdf]

Article DOI: http://dx.doi.org/10.3201/eid1901.121167

Novel Epidemic Clones of *Listeria monocytogenes*, United States, 2011

Technical Appendix

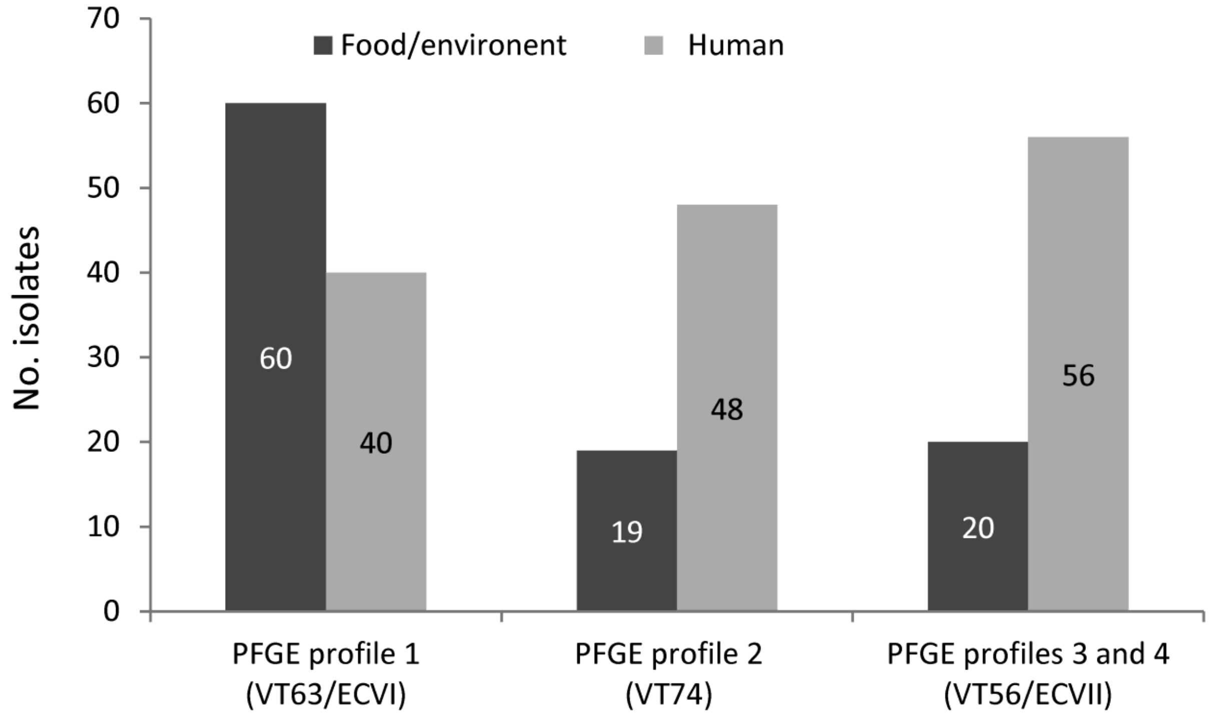

Technical Appendix Figure. Number of isolates of *Listeria monocytogenes* encountered in clinical and food or environment samples collected by the Centers for Disease Control and Prevention during a 2011 *L. monocytogenes* outbreak related to cantaloupe, which are representative of the 4 pulsed-field get electrophoresis (PFGE) profiles (identified at the time the research was performed) associated with the outbreak analyzed in the current study. PFGE profiles 3 and 4 were combined because they were the same virulence type (VT)56 and proposed epidemic clone (EC)VII.
